# Culture and affect: the factor structure of the affective style questionnaire and its relation with depression and anxiety among Japanese

**DOI:** 10.1186/1756-0500-7-590

**Published:** 2014-09-02

**Authors:** Masaya Ito, Stefan G Hofmann

**Affiliations:** National Center for Cognitive Behavior Therapy and Research, National Center of Neurology and Psychiatry, Tokyo, Japan; Department of Psychology, Boston University, Boston, MA USA; Ogawa Higashi 4-1-1, Kodaira, Tokyo, 187-8511 Japan

**Keywords:** Affective style, Emotion regulation, Culture, Depression, Anxiety

## Abstract

**Background:**

Affective styles are assumed to be one of the underlying processes of depression and anxiety maintenance. However, little is known about the effect of depression and anxiety and the cultural influence of the factor structure. Here, we examined the cross-cultural validity of the Affective Style Questionnaire and its incremental validity for the influence on depression and anxiety.

**Methods:**

Affective Style Questionnaire was translated into Japanese using standard back-translation procedure. Japanese university students (*N* = 1,041) served as participants. Emotion Regulation Questionnaire, Acceptance and Action Questionnaire-II, Toronto Alexithymia Scale, Rumination and Reflection Questionnaire, Brief COPE, Self-Construal Scale, and Hospital Anxiety and Depression Scale were administered.

**Results:**

Exploratory and confirmatory factor analyses showed that the Affective Style Questionnaire comprised four factors: Concealing, Adjusting, Holding and Tolerating (CFI = .92, TLI = .90, RMSEA = .07). The measure’s convergent and discriminant validity was substantiated by its association with various emotion regulation measures. Regression analyses showed that negative influence of Adjusting, Holding, Reappraisal (β = -.17, -.19, -.30) and positive influence of Suppression (β = .23) were observed on depression. For anxiety, Adjusting and Reappraisal was negatively influenced (β = -.29, and -.18).

**Conclusions:**

Reliability and validity of the Affective Style Questionnaire was partly confirmed. Further study is needed to clarify the culturally dependent aspects of affective styles.

## Background

Recent affective neuroscience, psychopathology, and psychotherapy research has focused on the dysregulation of emotion as an underlying transdiagnostic process of depression and anxiety [1–7]. Emotion regulation refers to “the processes by which individuals influence which emotions they have, when they have them, and how they experience and express these emotions” [8]. While cognitive behavioral therapies (CBT) were originally developed from learning theory and cognitive theory [9], the application of modern CBT incorporates the findings of recent advances in emotion regulation research to improve the therapeutic strategy [5]. Research on the topic of emotion regulation could enhance the translation and utilization of the neuroscience findings to improve the intervention strategy of CBT.

Hofmann et al. [5] reviewed the existing findings of emotion regulation research and proposed the emotion dysregulation model of depressive and anxiety disorders. This theory assumes that the person’s affective style, which is influenced by the interaction between external or internal triggers and the individual’s diathesis, determines the experience of positive and negative affect; if certain affective styles contribute to the maladaptive regulation to maintain negative affect, emotional disorders, such as depression and anxiety, can occur. According to this theory, the most effective treatment strategies for mood and anxiety disorders are by (1) targeting emotion dysregulation by promoting adaptive emotion regulation strategies, (2) decreasing negative affect and increasing positive effect, and (3) promoting adaptive affective styles [5]. This theory assumes that a person’s affective style is of central importance in mood and anxiety disorders. Affective style refers to the inter-individual differences in emotional sensitivity and regulation [10]. Thus, it is a trait-like individual difference variable that determines a person’s habitual use of emotion regulation strategies to specific emotional experiences.

Based on the existing literature on the topic of emotion regulation and affective style, Hofmann and Kashdan [11] developed the Affective Style Questionnaire (ASQ) to measure this individual difference variable. Factor analyses, using two separate United States (US) samples of university students, provided a three-factor structure: concealing, adjusting, and tolerating. *Concealing* refers to the tendency to suppress or avoid intrapersonal and interpersonal emotions after the generation of the emotion. *Adjusting* refers to the ability to be aware of and utilize the emotion as information, and modulate the emotional experience and expression in response to the demands of a given context. Finally, *tolerating* reflects the tendency to respond to an emotion in a nondefensive manner, even if the negative (or positive) emotion leads to some degree of distress.

These affective styles are one of the processes targeted by modern CBT in the treatment for depression and anxiety. Because both concealing and emotional avoidance involve attempts to not experience an emotion, these two affective styles share the conceptual similarity. The goal of the exposure strategies in CBT is to prevent the manifestation of the avoidant pattern and to allow the patient to experience the entire range of emotional experiences [12–14]. One of the core aspects of adjusting is the utilization of emotion as important information in a given situation. Recently, emotion-focused transdiagnostic CBT [13] specifically educates patients about the functional importance of emotions and to increase their emotional awareness of their emotional experiences. The goal of Acceptance-and-Commitment Therapy, Dialectical Behavior Therapy, and various types of Exposure Therapies are to develop the clients’ ability to tolerate distressing emotions [12, 15, 16].

It is assumed that these therapeutic strategies reduce depression and anxiety by preventing maladaptive concealing styles and by fostering more adaptive adjusting and tolerating styles. Hence, these affective styles have been hypothesized to have a significant effect on depression and anxiety. In fact, suppression, which is a form of concealment, has positive effect on anxiety and depression in Western samples [17]. Although the findings for adjusting and tolerating are limited, both could be conceptualized as adaptive forms of emotion regulation [5]. Hence, we hypothesized that the subscales of ASQ would be related to the anxiety and depression symptoms.

However, the clinical importance of affective styles and findings regarding ASQ may be culturally-specific. Although separate factor analyses from two samples of US university students revealed the cross-validity of the three factor structure, these results may be limited to a US population and may not be apply to other cultures. To the best of the author’s knowledge, there is no study that examined this factor structure in the non-western population. Before examining the relationships of affective styles with depression and anxiety, it is needed to confirm the factor structure, reliability and convergent and discriminant validity in the non-western culture.

Some findings suggest that culture has a profound impact on the construal of the world view and the self, which could result in differences in the processing of emotion and brain function within and between individuals [18, 19]. For example, the degree of reappraisal and suppression and the relationship between these two emotion regulation strategies were different among samples obtained from 23 different countries [20]. In this study, participants from cultures that emphasized the maintenance of social order, such as Japan, tended to have higher scores on suppression than did those from cultures that emphasized individual autonomy. Moreover, reappraisal and suppression tended to be positively correlated in the former culture, and tended to negatively correlate in the latter culture [20]. Culture may posit the display rules of emotion [21], and this could influence the degree or function of emotion regulation. For example, it has been shown that the social consequences of suppression are dependent on the person’s cultural values [22].

Given these considerations, we conducted this study to examine the three purposes. First purpose is to examine the cross-validation of factor structure with a sample comprised of Japanese university students. Second purpose is to examine the convergent and discriminant validity of the ASQ with related and unrelated variables. Third, we examine the influence of affective styles on depression and anxiety.

For the convergent and discriminant validity, we hypothesized that the correlation pattern of ASQ subscales and other emotion regulation variables observed in the original validation study [11] would be replicated in the Japanese sample. Specifically, we expect that adjusting would positively correlate to reappraisal [23] and psychological flexibility [24], and negatively correlated to difficulty in identifying and describing feelings [25]. As mentioned, one aspect of adjusting is express emotion in accordance with the circumstances. This ability is considered to be required to reappraisal and flexibility. Moreover, the ability to use emotion as information, which is other part of adjusting, requires the identifying and describing emotion without engaging in rumination. For concealing, we expected that this variable would be positively correlated to suppression [26] because of the conceptual commonality [11]. Tolerating would be positively correlated to psychological flexibility because it is suggested that one aspect of psychological flexibility is accepting emotion. In addition to these hypotheses, we also examined the relationship with the Self-Construal Scale (SCS; [27] to exploratory examine the cultural aspects of the ASQ. We did not set the hypotheses a priori for the relation between ASQ and SCS.

## Methods

### Participants

A total of 1,041 Japanese university students (540 females) from five Tokyo metropolitan area universities participated in this study (Nihon University = 222, Ochanomizu University = 42, Tokyo Seitoku College = 313, Tokyo University of Agriculture = 365, Rissho University = 99). These universities were all of available universities for the authors at the date of survey.

### Procedure and ethics

This study was approved by the Institutional Review Board of the National Center of Neurology and Psychiatry, Japan (Approved Number: A2010-028). A packet of anonymous questionnaires was administered after lectures at the universities. Before administering the survey, one of the authors or the instructor explained the purpose of the study and addressed any ethical issues. We also distributed documents describing the purpose of the study and related ethical issues before distributing the questionnaires. Participants gave informed consent by completing and submitting the questionnaires. Time One and Time Two data were matched by referring to the free key words written by each participant. No reward or course credit was given in exchange for participating in this study, which took approximately 20 minutes to complete.

The survey was conducted at two time points separated by one month. The participants from Nihon University and Ochanomizu University participated in the Time One but not the Time Two survey; answered demographic information questions; and completed the ASQ, the AAQ-II, the TAS, and the Brief COPE. Some participants from Tokyo Seitoku University, Tokyo University of Agriculture, and Rissho University participated in both, the Time One and Time Two surveys; other participated only in Time One or Time Two surveys. Participants answered demographic information questions, completed the ASQ (Time One, & Time Two), the ERQ (Time One), the SCS (Time One), the Rumination-Reflection Scale-shorter version (Time Two), and the Hospital Anxiety and Depression Scale (HADS) (Time Two).

### Measures

The original validation study of the ASQ examined the relationship of the ASQ with other emotion regulation measures, such as the Emotion Regulation Questionnaire (ERQ) [23], Berkeley Expressive Questionnaire [26], Acceptance and Action Questionnaire-II (AAQ-II [24], Toronto Alexithymia Scale (TAS; [25], Brief COPE ([28], and Difficulties in Emotion Regulation Scale [29]. Among them, we used the scales available in the Japanese language (i.e., ERQ, AAQ-II, TAS, Brief COPE) and Rumination and Reflection Questionnaire (Trapnel PD: RRQ shorter version, http://www.paultrapnell.com/measures/RRQ.pdf) that seemed conceptually related to ASQ.

#### ASQ

The ASQ [11] is a 20-item scale, measured on a 5-point Likert scale. Factor analyses using two different samples of US university students showed three factors: concealing, adjusting, and tolerating. The internal consistency values of the ASQ subscales in the US samples were .84, .80 − .82, and .66 − .68 for concealing, adjusting, and tolerating, respectively. The study demonstrated the convergent and discriminant validity in terms of the patterns of relations between existing instruments that measured similar constructs.

We used a standard back translation procedure [30] to translate the ASQ into Japanese. First, one of the Japanese authors translated the ASQ into Japanese. Second, a bilingual Japanese clinical psychologist, who has a master’s degree in psychology and did not know the original ASQ items, back-translated the Japanese version into English. Third, one of the original authors of the ASQ (SGH) checked the back-translated items for concordance of meaning and expression, and provided comments that specified the minor adjustments and modifications that had to be made to the wording of the questionnaire items. Fourth, the Japanese author modified some items and the Japanese bilingual psychotherapist again back-translated the items. Finally, the original author confirmed accuracy of the items.

#### ERQ

The ERQ [23] is a 10-item scale used to assess two types of emotion regulation: reappraisal and suppression. The items are scored on a 5-point Likert scale. Internal consistencies of the Japanese version were indexed by the Cronbach’s alpha values and were .77 and .78 for reappraisal and suppression, respectively [31]. Confirmatory factor analysis revealed that the Japanese version had a two-factor structure with weak positive relationship between reappraisal and suppression [31].

#### AAQ-II

The AAQ-II [24] is a 10-item scale used to assess emotional flexibility or experiential avoidance. Items are scored on a 5-point Likert scale. Sufficient internal consistency (Cronbach’s alpha = .86) and two week test-retest correlation (*r* = .62, *p* < .01) was reported in a study that used Japanese university students [32]. The authors also found evidence of convergent validity in terms of the instrument’s correlations with the trait subscale of the State-Trait Anxiety Inventory (*r* = -.72, *p* < .01), Zung Self-Reporting Depression Scale (*r* = -.50, *p* < .01), and White Bear Suppression Inventory (*r* = .62, *p* < .01). Higher questionnaire scores indicate higher emotional flexibility and lower emotional avoidance.

#### TAS

The TAS [25] is a 20-item scale used to measure three aspects of alexithymia: difficulty identifying feelings, difficulty describing feelings, and externally oriented thinking. The reliability and validity of the TAS has been demonstrated with a sample of Japanese participants [33]. Confirmatory factor analysis confirmed a three-factor structure with a Japanese sample. The Cronbach’s alpha values when used with a sample of Japanese university students were .85, .72, and .58 for difficulty identifying feelings, difficulty describing feelings, and externally oriented thinking, respectively.

#### Rumination and reflection questionnaire-shorter version

The Rumination and Reflection Questionnaire-Shorter Version (RRQ-S) is an 8-item measure, a short version of the original scale [34], used to assess rumination and reflection. The reliability and validity of the Japanese original version was confirmed [35]. The internal consistency in this study ranged from .68 (for reflection) to .78 (for rumination).

#### Brief COPE

The Brief COPE [28], an abbreviated version of the original scale [36], is a 28-item measure used to assess 14 types of stress-coping. Each subscale consists of two items, and the internal consistency (Cronbach’s alpha = .46–.80) of the subscales has been reported previously [37]. Although some of the subscales could be improved, a previous confirmatory factor analysis with a sample of Japanese participants showed an acceptable fit with the data [37].

#### SCS

The SCS [27] is a 31-item measure of two aspects of self-construal: interdependent self-construal and independent self-construal. The Japanese version of the SCS was provided by the original author. It is assumed that people in Eastern cultures tend to exhibit interdependent self-construal, whereas those in Western cultures tend to exhibit independent self-construal. The index of internal consistency in this study was .78 for both interdependent and independent self-construal.

#### HADS

The HADS [38] is a 14-item scale used to assess the severity of anxiety and depression. A previous confirmatory factor analysis, which confirmed the two-factor structure with a sample of Japanese participants, suggested a sufficient fit with the data. The reliability and validity of the Japanese version has been reported previously [39]. The internal consistency coefficients for the Japanese version were .76 and .81 for depression and anxiety, respectively [39].

### Statistical analyses

First, we used the data obtained at Time One to conduct an exploratory factor analyses (EFA) to examine the factor structure of the ASQ. The original validation study [11] employed principal components extraction with varimax rotation. We used this method to determine whether the original three-factor structure could be replicated with the data from the present study. The number of appropriate factors was determined by examining the Eigen values, the scree test, and the interpretability of the factors. After the first EFA, we excluded the items that cross-loaded onto more than one factor (loadings > .35), and repeated the same EFA without these items. This factor analysis procedure was based on the recommendation provided by Costello and Osborne [40]. Next, the data obtained at Time Two were used in a confirmatory factor analysis to determine and cross-validate the factor structure in this sample of Japanese participants. Goodness-of-fit indices, including the root mean square error of approximation (RMSEA), the Comparative Fit Index (CFI), and the Tucker–Lewis Index (TLI) were examined to determine how well the model fit the data (D’Avanzato et al.). Standardized factor loadings were used to assess the appropriateness of the measurement for the latent factor.

The reliability of the ASQ was determined by calculating Cronbach’s alpha and test-retest correlation coefficients. Means and standard deviations of the ASQ subscales were calculated for the scores obtained at Time One and Two. The correlations between the subscales of the ASQ and various measures (i.e., the ERQ, AAQ-II, TAS, COPE, RRQ-S, and SCS) were calculated to examine the ASQ’s convergent and discriminant validity. Then, we performed multiple regression analyses to examine the influence of the subscales of the ASQ and ERQ at Time one on depression and anxiety at Time two (i.e., one month after). We included the ERQ in the regression analysis because the majority of the studies of emotion regulation have been based on the process model of emotion regulation proposed by Gross [8] and used the ERQ. All *p* values were two-tailed. Statistical analyses were conducted using PASW Statistics for Windows, Version 18.0 (Chicago; SPSS Inc) and Amos 18.0 [41].

## Results

### Factor structure of the ASQ

The mean age of the participants was 19.89 years (*SD* = 1.37, range = 18–29). Both, the overall Kaiser-Meyer-Olkin index of sampling adequacy (.89) and Bartlett’s test of sphericity (χ^2^(190) = 5607.03, *p* < .001) suggested that the data were appropriate for a factor analysis. An EFA, using principal components extraction with varimax rotation, was performed on the ASQ, using the data obtained at Time One. Five factors had Eigen values greater than or equal to 1.00: 6.18, 2.33, 1.22, 1.15, and 1.00. A close inspection of the factor structure suggested that the items that loaded on the first four factors described four distinct affective styles: concealing, adjusting, holding, and tolerating. Four items (concealing #8, adjusting #2, adjusting #6, tolerating #4 in the original article) loaded onto more than one factor (loading > .35). These four items were eliminated from the subsequent EFA. Four factors were extracted from the subsequent EFA (Table 1). The first factor (concealing), consisting of 6 items, accounted for 20.66% of the variance; the second factor (adjusting), consisting of 4 items, accounted for 16.63% of the variance; the third factor, consisting of 3 items, accounted for 11.47% of the variance, was interpreted as holding because the content of the items reflected the holding emotion; finally, the fourth factor (tolerating), consisting of 3 items, accounted for 9.17% of the variance. All items loaded onto only one factor (loadings > .54) and did not cross-load onto the other factors (loadings < .35).

**Table 1:** **The factor loadings obtained by the exploratory factor analysis of the ASQ**

Items	Concealing	Adjusting	Holding	Tolerating
I am good at hiding my feelings (CON #3)	**.76**	.11	.28	.03
I can act in a way that people don’t see me being upset (CON #6)	**.75**	.22	.17	.04
People usually can’t tell when I am upset (CON #4)	**.74**	.16	.11	-.14
People usually can’t tell when I am sad (CON #5)	**.73**	.10	.03	.02
I could easily fake emotions (CON #7)	**.70**	.10	.06	.22
People usually can’t tell how I am feeling inside (CON #1)	**.54**	-.01	.26	.01
I am able to let go of my feelings (ADJ #4)	.07	**.85**	.03	-.05
I can get out of a bad mood very quickly (ADJ #5)	.12	**.79**	.06	.24
I can calm down very quickly (ADJ #3)	.19	**.71**	.21	.08
I can get into a better mood quite easily (ADJ #7)	.14	**.66**	.09	.24
I can tolerate having strong emotions (TOL #1)	.18	.16	**.82**	.09
I have my emotions well under control (ADJ #1)	.13	.34	**.67**	.15
I often suppress my emotional reactions to things (CON #6)	.34	-.07	**.67**	-.08
There is nothing wrong with feeling very emotional (TOL #5)	.08	-.05	.13	**.75**
It’s ok to feel negative emotions at times (TOL #3)	.20	.32	.02	**.61**
It’s ok if people see me being upset (TOL #2)	-.14	.19	-.03	**.54**

Factors that loaded in the U. S. sample indicated in parentheses.

ASQ, Affective Style Questionnaire; CON, Concealing; ADJ, Adjusting; TOL, Tolerating.

Bold numbers mean the factor loadings were above .50.

In order to confirm the factor structure obtained in the EFA, a confirmatory factor analysis was conducted, using the data collected at Time Two. The goodness of fit indices were adequate [χ^2^ = 349.55, *df* = 98, χ^2^/*df* = 3.57, CFI = .92, TLI = .90, RMSEA = .07 (90% CI: .08 − .06)]. The standardized loadings were at least moderately high (loadings > .49) except for two items on the factor, tolerating (tolerating #2 = .35 and tolerating #5 = .39).

### Psychometric data

Means, standard deviations, and Cronbach’s alphas for each of the ASQ subscales measured at Time One and Time Two are shown in Table 2. The test-retest correlations were as follows: concealing (*r* = .73, *p* < .001), adjusting (*r* = .76, *p* < .001), holding (*r* = .66, *p* < .001), and tolerating (*r* = .60, *p* < .001).

**Table 2:** **The means, standard deviations and Cronbach’s alpha values for the ASQ subscales**

	Time one			Time two		
	Mean	***SD***	alpha	Mean	***SD***	Alpha
Concealing	17.59	4.97	.83	17.68	5.08	.87
Adjusting	10.51	3.66	.80	10.64	3.63	.83
Holding	9.44	2.53	.67	9.40	2.50	.73
Tolerating	9.52	2.29	.42	9.50	2.28	.47

### Convergent and discriminative validity of the ASQ

We calculated correlations between the three affective styles obtained from the factor analyses (mentioned in the previous sections) with the various measures of emotion regulation (Table 3). As expected, concealing was positively correlated with suppression (*r* = .54, *p* < .001) and negatively correlated with emotional and instrumental support (*r* = -.26 and -.28, respectively, *p* < .001). Adjusting was positively correlated with reappraisal and the AAQ-II (*r* = .39 and .51, *p* < .001), and negatively correlated with rumination (*r* = -.44, *p* < .001). Holding was positively correlated with suppression and reappraisal (*r* = .46 and .28, respectively, *p* < .001), and negatively correlated with emotional and instrumental support (*r* = -.26 and -.28, respectively, *p* < .001). Although the pattern of correlations were similar between concealing and holding, only holding was positively correlated with the AAQ-II and positive reframing (*r* = .19 and .17, respectively, *p* < .001), and negatively correlated with substance use and venting, although the correlations were relatively small (*r* = -.16 and -.22, respectively, *p* < .05).

**Table 3:** **The Correlations between the affective styles and the other instruments**

	Concealing		Adjusting		Holding		Tolerating	
ASQ								
Concealing	-		.33	**	.49	**	.16	**
Adjusting	-		-		.33	**	.37	**
Holding	-		-		-		.19	**
Tolerating	-		-		-		-	
ERQ								
Reappraisal	.26	**	.39	**	.28	**	.15	**
Suppression	.54	**	.17	**	.46	**	.01	
AAQ-II	.13	*	.51	**	.19	**	.23	**
TAS-20								
Difficulty identifying feelings	-.03		-.33	**	-.06		-.13	*
Difficulty describing feelings	.08		-.25	**	-.01		-.16	**
Externally oriented	.17	**	.05		.13	*	-.06	
RRQ-shorter version								
Rumination	-.11		-.44	**	-.16	**	-.15	*
Reflection	.15	*	-.03		.06		.04	
Brief COPE								
Self-Distraction	-.03		.16	**	-.10		.00	
Active Coping	.03		.20	**	.11		.12	*
Denial	.01		-.05		-.02		-.01	
Substance Use	-.02		-.09		-.16	*	-.19	**
Emotional Support	-.26	**	-.09		-.26	**	.02	
Instrumental Support	-.28	**	-.02		-.27	**	-.01	
Behavioral Disengagement	.17	**	-.14	*	.01		-.07	
Venting	-.10		-.10		-.22	**	.02	
Positive framing	.02		.24	**	.17	**	.14	*
Planning	.06		.18	**	.11		.06	
Humor	.11		.20	**	.03		.05	
Acceptance	.03		.20	**	.14	*	.16	**
Religion	-.07		-.11		-.10		.01	
Self-Blame	.03		-.31	**	.01		-.12	
SCS								
Independent self-construal	.16	**	.31	**	.08		.28	**
Interdependent self-construal	.07		.06		.17	**	.10	*

ASQ Affective Style Questionnaire, ERQ Emotion Regulation Questionnaire, AAQ-II Acceptance and Action Questionnaire-II, TAS Toronto Alexithymia Scale, RRQ-shorter version Rumination and Reflection Questionnaire-shorter version, SCS Self-Construal Scale.

**p < .01, *p < .05.

### Cultural construal of self and affective styles

We calculated the correlations between affective styles and cultural construal of self (Table 3). Independent self-construal was correlated with concealing (*r* = .16, *p* < .001) and adjusting (*r* = .31, *p* < .001), whereas interdependent self-construal was correlated with holding (*r* = .17, *p* < .001).

### Incremental validity of the ASQ for the influence on depression and anxiety

Multiple regression analyses to examine the relation with depression and anxiety were conducted. Three types of affective styles (concealing, adjusting, and holding) and ERQ subscales (reappraisal and suppression) were entered simultaneously into the regression analyses. Because the tolerating factor showed poor internal consistency (Cronbach’s alpha = .42–.47), we excluded it from these regression analyses. The results of these analyses are presented in Table 4.

**Table 4:** **Summary statistics for the regression analyses on depression and anxiety of the ASQ and the ERQ**

Variable	***R*** ^***2***^	***β***	***t***	***p***
Depression				
Overall model	.21			
ASQ Concealing		-.01	−0.21	.837
ASQ Adjusting		-.17	−2.77	.006
ASQ Holding		-.19	−2.98	.003
ERQ Reappraisal		-.30	−5.02	.000
ERQ Suppression		.23	3.34	.001
Anxiety				
Overall model	.17			
ASQ Concealing		.04	0.50	.616
ASQ Adjusting		-.29	−4.58	.000
ASQ Holding		-.11	−1.59	.112
ERQ Reappraisal		-.18	−2.90	.004
ERQ Suppression		.12	1.76	.079

ASQ Affective Style Questionnaire, ERQ Emotion Regulation Questionnaire.

## Discussion

The purposes of this study were to (1) conduct a cross-validation of factor structure of the ASQ in a Japanese university sample, (b) to examine the reliability, convergent and discriminant validity of the ASQ subscales, and (c) to examine the influence of affective styles on depression and anxiety in a Japanese sample. Some of our findings describing the factor structure differed from those of the original study, which used a sample of American participants. Consistent with the factors obtained in the US study, we also identified the concealing, adjusting, and tolerating factors. However, the tolerating factor had poor reliability. Moreover, a new factor, labeled *holding*, emerged. Correlation analyses indicated that these subscales had acceptable convergent and discriminant validity. Regression analyses showed that, in addition to the conventional measure of emotion regulation (reappraisal and suppression), the factor adjusting was negatively associated with depression and anxiety, and the factor holding was negatively associated with depression. These results underscore the importance of focusing on other types of affective styles that have not been systematically examined by previous studies and that may be culture specific.

Our factor analyses, using the same method and rotation as that described in the original paper, produced a four-factor structure when applied to a sample of Japanese participants. The concealing, adjusting, and tolerating factors consisted of the items that loaded onto the same factor as described in the original paper. In addition to these replicated factors, a new factor, labeled *holding*, was obtained. This factor consisted of three items that originally loaded onto the factors concealing, adjusting, and tolerating. This factor appears to reflect the ability to control emotions through self-restrain. It further appears that the holding strategy is different from simply tolerating and emotion. Although the test-retest correlation coefficient of tolerating was acceptable, the internal consistency was poor. Because this subscale consisted of only three items, one way of improving its reliability might be developing and adding other items reflecting the meaning of tolerating. In terms of test-retest reliability, none of the four factors of the subscale could not be concluded as stable (*r* = .60 − .76). These results mean only 36–58% of variance could be explained by the score at the time of one month ago. This finding suggests that the affective style could vary over time and, hence, be targeted for enhancement or prevention by CBT.

The affective styles (concealing, adjusting, tolerating, and holding) were weakly or moderately correlated with each other. Thus, all three styles might reflect some degree of regulating emotion. Interestingly, adjusting was moderately correlated (*r* = ~.35) with the other three styles. Thus, adjusting could be an important regulation style that is involved in many emotion regulation types. For example, it might be needed to adjust emotion in order to tolerate, suppress, or hold.

Convergent and discriminant validity is generally supported for ASQ subscales. The correlational data for the ASQ subscales of concealing and adjusting were consistent with the previous findings of studies conducted in the US. Adjusting was correlated with the AAQ-II (*r* = .51), reappraisal (*r* = .39), difficulty identifying feelings (*r* = -.33), and difficulty describing feelings (*r* = -.25). These results support the conceptualization of adjusting as the tendency for emotional awareness and to utilize emotion as information important for guiding behavior. The finding of a moderate correlation between concealing and suppression (*r* = .54) was also consistent with the results of those that used a sample of American participants. Whereas concealing was found to be positively correlated with difficulty identifying feelings (*r* = .18) and with difficulty describing feeling (*r* = .38) in the earlier US study, the correlations were not significant in the present study, which used a sample of Japanese participants. Thus, concealing might not be as maladaptive in an Asian culture as it is in a Western culture [22]. Holding, a new subscale identified in the present study, seems to have a similar meaning as concealing. In fact, the moderate relationship found between these affective styles (*r* = .49, *p* < .01) was similar to the pattern of the relationship with other instruments. However, only holding was negatively correlated with venting coping and positively correlated with positive reframing coping. These results suggest that holding might be a more adaptive style of regulating emotions in the Japanese culture.

Concealing and adjusting were positively correlated with independent self-construal, and holding was positively correlated with interdependent self-construal. Along with the results of the factor analyses, these results imply that holding is one of the unique styles of emotion regulation used in an Asian culture.

Our regression analyses showed the importance of focusing on affective style in considering its adaptive or maladaptive function in emotion regulation. Specifically, reappraisal, holding, and adjusting were negatively associated with depression, whereas suppression was positively associated with depression. These reappraisal and suppression findings were consistent with the previous findings [17]. Moreover, holding and adjusting were negatively associated with depression. In the correlational analyses, both affective styles were negatively correlated with rumination. Hence, these affective styles might have negative association with depression through the mediating role of rumination. Holding is also similar to distress tolerance. As distress tolerance is associated with low depression [42], this factor may contribute to the inhibitory effect of holding on depression. Adjusting and reappraisal were associated with low anxiety. As with the depression findings, the reappraisal finding is in line with the existing literature [17]. Interestingly, adjusting had a stronger negative association with anxiety. These results support the view that emotional awareness and emotion utilization play important roles in the treatment of anxiety disorders [13].

These results, however, must be evaluated while considering the limitations of this study. First and foremost, the participants were university students living in an urban area of Japan. Consequently, we do not know if the results of this study generalize to clinical populations, such as those suffering from depression or an anxiety disorder. Moreover, because affective styles seem to play a key role in the maintenance of such disorders [5], it is necessary to replicate the present findings by using clinical samples. Second, though we employed the prospective design to examine the influence of ASQ on depression and anxiety, the time interval was relatively short (i.e., one month). Third, we did not have a priori hypothesis for the cultural difference of factor structure or other findings regarding ASQ. Holding, the new factor obtained in this study, might be culturally specific affective style for Japanese, or Asian culture. Further study should be conducted to examine this factor conceptually and psychometrically.

## Conclusions

Despite of these limitations, this study provides the first findings on factor structure of the ASQ and its relation with depression and anxiety among Asian population. This study adds important information to the expanding field of emotion regulation research by highlight the importance of considering cultural factors, which may lead to novel affective styles (i.e., holding) associated with depression and anxiety.

## Abbreviations

ASQ: Affective style questionnaire
CBT: Cognitive behavioral therapy
ERQ: Emotion regulation questionnaire
AAQ-II: Acceptance and action questionnaire-II
TAS: Toronto Alexithymia scale
SCS: Self-construal scale
RRQ-S: Rumination and reflection questionnaire-shorter version
HADS: Hospital anxiety and depression scale
EFA: Exploratory factor analyses
RMSEA: Root mean square error of approximation
CFI: Comparative fit index
TLI: Tucker-lewis index.
